# An internal deletion in *MTH1* enables growth on glucose of pyruvate-decarboxylase negative, non-fermentative *Saccharomyces cerevisiae*

**DOI:** 10.1186/1475-2859-11-131

**Published:** 2012-09-15

**Authors:** Bart Oud, Carmen-Lisset Flores, Carlos Gancedo, Xiuying Zhang, Joshua Trueheart, Jean-Marc Daran, Jack T Pronk, Antonius JA van Maris

**Affiliations:** 1 Department of Biotechnology, Delft University of Technology, Julianalaan 67, 2628 BC, Delft, The Netherlands; 2 Kluyver Centre for Genomics of Industrial Fermentation, P.O. Box 5057, 2600 GA, Delft, The Netherlands; 3 Department of Metabolism and Cell Signaling, Instituto de Investigaciones Biomédicas “Alberto Sols” CSIC-UAM, 28029, Madrid, Spain; 4 Microbia Inc., 60 Westview Street, Lexington, MA, 02421, USA; 5 Present address: Merrimack Pharmaceutical Inc., One Kendall Square, Suite B7201, Cambridge, MA, 02139, USA

**Keywords:** Inverse metabolic engineering, Reverse metabolic engineering, Whole genome sequencing, Glucose tolerance, by-product reduction, *MTH1* allele

## Abstract

**Background:**

Pyruvate-decarboxylase negative (Pdc^-^) strains of *Saccharomyces cerevisiae* combine the robustness and high glycolytic capacity of this yeast with the absence of alcoholic fermentation. This makes Pdc^-^*S. cerevisiae* an interesting platform for efficient conversion of glucose towards pyruvate-derived products without formation of ethanol as a by-product. However, Pdc^-^ strains cannot grow on high glucose concentrations and require C_2_-compounds (ethanol or acetate) for growth under conditions with low glucose concentrations, which hitherto has limited application in industry.

**Results:**

Genetic analysis of a Pdc^-^ strain previously evolved to overcome these deficiencies revealed a 225bp in-frame internal deletion in *MTH1*, encoding a transcriptional regulator involved in glucose sensing. This internal deletion contains a phosphorylation site required for degradation, thereby hypothetically resulting in increased stability of the protein. Reverse engineering of this alternative *MTH1* allele into a non-evolved Pdc^-^ strain enabled growth on 20 g l^-1^ glucose and 0.3% (v/v) ethanol at a maximum specific growth rate (0.24 h^-1^) similar to that of the evolved Pdc^-^ strain (0.23 h^-1^). Furthermore, the reverse engineered Pdc^-^ strain grew on glucose as sole carbon source, albeit at a lower specific growth rate (0.10 h^-1^) than the evolved strain (0.20 h^-1^). The observation that overexpression of the wild-type *MTH1* allele also restored growth of Pdc^-^*S. cerevisiae* on glucose is consistent with the hypothesis that the internal deletion results in decreased degradation of Mth1. Reduced degradation of Mth1 has been shown to result in deregulation of hexose transport. In Pdc^-^ strains, reduced glucose uptake may prevent intracellular accumulation of pyruvate and/or redox problems, while release of glucose repression due to the *MTH1* internal deletion may contribute to alleviation of the C_2_-compound auxotrophy.

**Conclusions:**

In this study we have discovered and characterised a mutation in *MTH1* enabling Pdc^-^ strains to grow on glucose as the sole carbon source. This successful example of reverse engineering not only increases the understanding of the glucose tolerance of evolved Pdc^-^*S. cerevisiae*, but also allows introduction of this portable genetic element into various industrial yeast strains, thereby simplifying metabolic engineering strategies.

## Background

*Saccharomyces cerevisiae* continues to prove its potential as an excellent microbial production platform of many bulk chemicals [1-4]. While traditionally *S. cerevisiae* has mainly been used for its high speed and capacity to convert sugars into ethanol and CO_2_, presently its robustness and genetic accessibility are also much appreciated in many metabolic engineering efforts for production of bio-based fuels [5-7] and chemicals [8-11]. In fact, in several industrial processes, including those centered around pyruvate-derived products such as malate [12,13] or lactate [14-16], ethanol is now considered an undesired by-product.

Even under fully aerobic conditions, *S. cerevisiae* converts part of its sugar substrate to ethanol when confronted with high sugar concentrations [17]. Conversion of glucose to ethanol yields much less ATP than complete conversion to CO_2_ and H_2_O via respiratory dissimilation, which is a drawback in ATP-requiring production processes [18]. The strong tendency of *S. cerevisiae* towards alcoholic fermentation is thought to have evolved as a mechanism to outcompete other organisms by the resulting fast glucose uptake and build-up of growth-inhibiting ethanol concentrations [19,20]. Although beneficial in natural environments, in many applied contexts this phenomenon lowers product yields. Therefore, several metabolic engineering studies have sought to disrupt aerobic fermentation of sugars by *S. cerevisiae*[21-26].

A powerful approach to prevent alcoholic fermentation in *S. cerevisiae* is elimination of pyruvate decarboxylase, which catalyzes the first step in the conversion of pyruvate to ethanol. *S. cerevisiae* strains in which all three structural genes encoding pyruvate decarboxylase (*PDC1PDC5* and *PDC6*[27]) were deleted, did not produce ethanol, but were unable to grow in the presence of high glucose concentrations and, when grown in glucose-limited cultures, required the addition of ethanol or acetate to growth media, due to their inability to synthesize cytosolic acetyl-CoA from pyruvate [23-25]. To overcome these deficiencies, a Pdc^-^ yeast was selected for growth on glucose as the sole carbon source in an evolutionary engineering experiment [25]. First, C_2_-carbon source prototrophic mutants were selected by prolonged cultivation in glucose-limited chemostat cultures, in which the acetate concentration in the medium gradually decreased to zero. Subsequently, a mutant able to grow at high glucose concentrations was selected by cultivation in serial shake flask cultures. The resulting evolved mutant could grow at a growth rate of 0.20 h^-1^ on synthetic medium with glucose as the sole carbon source and proved to be an efficient pyruvate producer [25].

Elucidation of the genetic background of glucose tolerance in Pdc^-^*S. cerevisiae* is not only of fundamental interest, but is also required to enable its fast introduction in metabolic engineering strategies. The process of elucidating and subsequent reconstruction of a desired phenotypic trait is known as reverse metabolic engineering [28,29]. Reverse engineering of phenotypes obtained by laboratory evolution has the added benefit that potential detrimental effects of random mutations obtained during evolution can be eliminated. Identification of relevant mutations is an essential step in reverse metabolic engineering. Transcriptional profiling of the evolved Pdc^-^ mutant during growth in nitrogen-limited chemostat cultures revealed the altered expression of many hexose transporters (Hxt) in this evolved strain compared to a wild type strain [25]. It was found that the summed transcript abundance of all HXT genes represented on the arrays (*HXT1* to *HXT10HXT12HXT14*, and *HXT16*) was four-fold lower in the evolved Pdc^-^ strain than in a Pdc^+^ reference strain [25].

Transcription of *HXT* genes in *S. cerevisiae* is predominantly regulated via the transcriptional regulator Rgt1 [30-33], which also regulates *MIG2* and *STD1* expression [34-36]. *MIG2* and *STD1* are both down-regulated in the evolved Pdc^-^ strain [25]. Rgt1 is regulated by the concerted action of the glucose sensors Rgt2 and Snf3, which relay the extracellular glucose signal via the paralogous repressors Mth1 and Std1 to Rgt1 [33,36-40]. In the absence of extracellular glucose, Mth1 and Std1 are in a complex with Rgt1, Ssn6 and Tup1 resulting in the transcriptional repression or activation of Rgt1 targets [41-43]. In the presence of glucose, the conformation of the glucose sensors Rgt2 and Snf3 is thought to change, which facilitates the phosphorylation of Mth1 and Std1 by Yck1 [38]. When phosphorylated, Mth1 and Std1 are targeted for degradation [38]. The absence of Mth1 or Std1 enables phosphorylation of Rgt1 [30,42,44], which is subsequently released from the promoters of, amongst others, the Hxt transporters [30-33]. The altered transcript profiles of *HXT* genes in the evolved, glucose-tolerant Pdc^-^*S. cerevisiae* strain might therefore be explained by mutations in this regulatory network. For a comprehensive review and graphical representation of the regulation of the *HXT* transporters see Gancedo et al. 2008 [36].

The goal of the present study was to identify the mutation(s) responsible for the ability of the evolved Pdc^-^ strain isolated by Van Maris *et al*. (2004) to grow on high concentrations of glucose as sole carbon source. Our results identified a mutation in *MTH1,* whose impact on growth on glucose in the absence of added C_2_-compounds was investigated after reintroduction in an ancestral Pdc^-^*S. cerevisiae.*

## Results

### An evolved Pdc^-^*S. cerevisiae* strain has an internal deletion within *MTH1*

To investigate the genetic basis of the ability of the evolved Pdc^-^*S. cerevisiae* strain (TAM) to grow at high glucose concentrations, the strain was crossed with a *pdc1 pdc5* strain of the opposite mating type (*pdc1 pdc5* strains are unable to grow on glucose despite the presence of the weakly expressed *PDC6* gene [23]). The resulting diploid was able to grow on 20 g l^-1^ glucose, indicating that the glucose tolerance of the TAM strain is a dominant trait. Upon sporulation of this diploid and dissection on YP medium supplemented with 2% ethanol (v/v), only one of 23 tetrads yielded four viable spores, twelve yielded three, six yielded two, and four yielded one. Fifty-two spores from the nineteen tetrads with more than one viable spore were tested for growth on YPD, and it was found that 28 were strongly glucose-tolerant, six were weakly so, and eighteen were glucose-sensitive. These results, coupled with the observation that no tetrad yielded more than two glucose sensitive segregants, strongly suggested that the trait is monogenic. PCR analysis of the segregants showed that *PDC6* segregated independently from the glucose tolerant phenotype.

**Figure 1:**
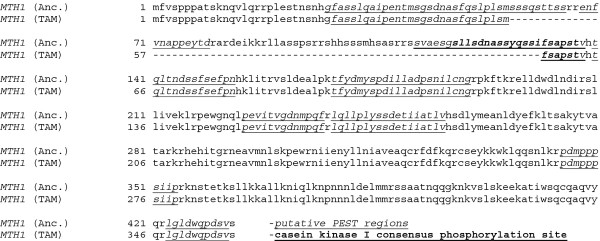
**Alignment of the Mth1 amino acid sequences from TAM and RWB837.** TAM is a Pdc^-^ strain evolved for growth on glucose and RWB837 is the ancestral Pdc^-^ strain from which the TAM strain was derived. The casein kinase I consensus phosphorylation site is underlined and bold. The deleted region is rich in amino acid stretches in serine and other amino acids which may form PEST regions that are associated with proteins that have short half-lives [50,53].

Several alleles of *MTH1*, which encodes a transcriptional regulator involved in glucose sensing, are known to dominantly suppress the glucose sensitivity of several other glucose-intolerant mutants [32,45-49]. A hypothesis to explain the glucose tolerance of the evolved Pdc^-^ strain TAM could be that it is caused by a mutation in the *MTH1* gene. This would be consistent with the previously observed transcriptional changes of *HXT* genes in nitrogen-limited, glucose-grown chemostat cultures [25]. Sequencing of the 1302 basepairs of the *MTH1* ORF from the evolved Pdc^-^ strain revealed a 225 bp internal deletion spanning from position 169 to 393 of *MTH1* (Figure 1). This new allele was named *MTH1-ΔT*. The deletion found in *MTH1-ΔT* does not disrupt the reading frame but it affects two important characteristics of the protein. Firstly, it eliminates a sequence phosphorylated by the yeast casein kinase Yck1, required for degradation of Mth1 [38] (Figure 1). Secondly, it removes amino acid stretches rich in serine and other amino acids which may form PEST regions that are associated with proteins that have short half-lives [50].

### Introduction of *MTH1-ΔT* in the ancestral Pdc^-^ strain restores growth on glucose

If the mutation in *MTH1* found in the evolved Pdc^-^ strain is responsible for its glucose tolerant phenotype, substitution of the chromosomal wild-type allele of *MTH1* in a non-evolved Pdc^-^ strain should render it glucose tolerant. This was experimentally tested by integration of a cassette containing *MTH1-ΔT* in the *MTH1* locus of RWB837 and subsequent selection of uracil-auxotrophic revertants with only the *MTH1-ΔT* allele. After confirmation of correct integration, this yielded strain IMI076 (Pdc^-^*MTH1-ΔTura3*). Since quantitative growth studies are best performed using prototrophic strains [51], the *URA3* gene was subsequently repaired, resulting in strains IMI078 (Pdc^-^*MTH1-ΔT*), IMI082 (Pdc^-^*MTH1*) and IMI083 (evolved Pdc^-^).

**Figure 2:**
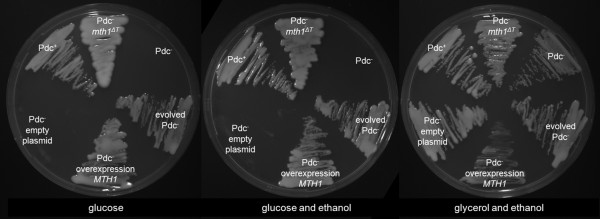
**Growth of*S. cerevisiae*strains with different*MTH1*alleles on synthetic medium agarose plates with 20 g l**^**-1**^**glucose as the sole carbon source (left plate), 20 g l**^**-1**^**glucose supplemented with 0.3% (v/v) ethanol (middle plate) or 2% (v/v) glycerol and 0.3% (v/v) ethanol (right plate).** The strains used were: IMI078 (Pdc^-^*MTH1-ΔT*), IMI082 (Pdc^-^), IMI083 (evolved Pdc^-^), IMZ104 (Pdc^-^ overexpression *MTH1*), IMZ103 (Pdc^-^ empty plasmid), CEN.PK113-7D (Pdc^+^ reference). Plates were incubated at least 3 days at 30**°**C.

As expected for Pdc^-^*S. cerevisiae*, all strains grew on the positive-control plates containing glycerol and ethanol as the carbon source (Figure 2). As previously reported, the parental Pdc^-^ strain (IMI082 (Pdc^-^*MTH1*)) did not grow in the presence of 20 g l^-1^ glucose, whereas the evolved Pdc^-^ strain (IMI083 (evolved Pdc^-^)) did grow. The unevolved Pdc^-^ strain carrying the *MTH1-ΔT* allele (IMI078 (Pdc^-^*MTH1-ΔT*)), grew equally well as the evolved Pdc- strain on plates with 20 g l^-1^ glucose supplemented with 0.3% (v/v) ethanol as C_2_-source, thereby demonstrating that the internal deletion in *MTH1* is sufficient to confer glucose tolerance to Pdc^-^*S. cerevisiae*.

If the deletion of the phosphorylation site in *MTH1-ΔT* resulted in decreased degradation of the Mth1 protein and thereby in an increased abundance of Mth1 in the cell, direct overexpression of the native *MTH1* might also confer glucose tolerance to a Pdc^-^ strain. To challenge this hypothesis, the native *MTH1* gene was expressed from the strong *PGK1* promoter on a multicopy plasmid in the ancestral Pdc^-^ strain RWB837 (yielding strain IMZ104). The transformed strain grew on agarose plates with 20 g l^-1^ glucose and 0.3% (v/v) ethanol, whereas the empty plasmid control (strain IMZ103) did not grow (Figure 2).

### Characterization of evolved Pdc^-^ and reverse engineered *MTH1-ΔT* Pdc^-^*S. cerevisiae* in bioreactors

**Figure 3:**
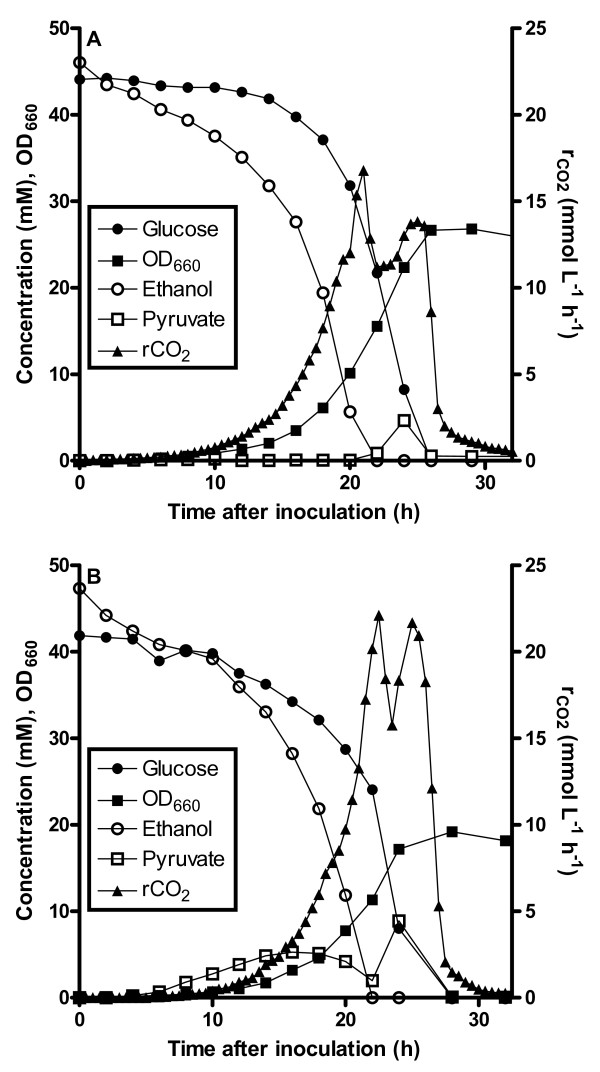
**Growth profile of IMI078 (A; Pdc**^**-**^***MTH1-ΔT*) and IMI083 (B; evolved Pdc**^**-**^**) on high concentrations of glucose.** Cultivation in an aerated pH-controlled (pH 5) bioreactor with 7.5 g l^-1^ glucose and 0.3% (v/v) ethanol. The results are from one representative experiment. Duplicate experiments deviated <5% in titers, CO_2_ production and OD_660_.

Although the characterization on plates provided a qualitative demonstration that the *MTH1-ΔT* allele confers glucose tolerance to Pdc^-^ strains, quantitative analysis of growth and product formation required cultivation under controlled conditions. Therefore, strains IMI078 (Pdc^-^*MTH1-ΔT*) and IMI083 (evolved Pdc^-^) were grown in aerobic, pH-controlled bioreactors on synthetic medium supplemented with 7.5 g l^-1^ glucose and 0.3% (v/v) ethanol (Figure 3). Under these conditions, the specific growth rates of strain IMI078 (Pdc^-^*MTH1-ΔT*; 0.24 ± 0.00 h^-1^) and strain IMI083 (evolved Pdc^-^; 0.23 ± 0.00 h^-1^) were virtually the same. The observation that reverse engineering of *MTH1-ΔT* resulted in near-identical specific growth rates in glucose-ethanol grown batch cultures as observed with the evolved Pdc^-^ strain, is consistent with the observation that the glucose tolerance was monogenic.

During the first growth phase, in which ethanol and glucose were simultaneously consumed, the yield of pyruvate on substrate was higher in the evolved strain (0.30 ± 0.04 g_pyr_ g_glc+etoh_^-1^) than in the reverse engineered strain IMI078 (Pdc^-^*MTH1-ΔT*; 0.07 ± 0.03 g_pyr_ g_glc+etoh_^-1^) (p-value = 0.02; student’s t-test; n = 2). Apparently, there is/are additional mutation(s) that affect the extracellular accumulation of pyruvate. Both Pdc^-^ strains showed a decrease of the rate of CO_2_ production when the ethanol added to the medium was depleted, which was caused by a decrease in the specific glucose consumption rate. Whilst the remaining glucose was consumed, the biomass concentration increased and as a consequence the volumetric CO_2_ production rate also increased again. After all the glucose was consumed, the volumetric CO_2_ production rate rapidly decreased while pyruvate, the main metabolite produced during the glucose consumption phase, was consumed (Figure 3).

In both strains, the optical density of the cultures increased by ca. 50 % after ethanol had been depleted. This result was expected for the evolved strain IMI083, which was specifically selected for its ability to grow on glucose in the absence of externally added C_2_-sources [25]. The biomass formation of strain IMI078 (Pdc^-^*MTH1-ΔT*) in this growth phase could either indicate a redistribution of lipids and lysine over newly synthesized cells or indicate that, in addition to increasing the glucose tolerance of Pdc^-^*S. cerevisiae*, presence of the *MTH1-*^*ΔT*^ allele had an additional impact on the C_2_-compound auxotrophy of Pdc^-^ strains. This observation is consistent with growth on plates with glucose as the sole carbon source.

### Introduction of the *MTH1-*^*ΔT*^ allele partially alleviates the C_2_-compound auxotrophy of Pdc^-^*S. cerevisiae*

**Figure 4:**
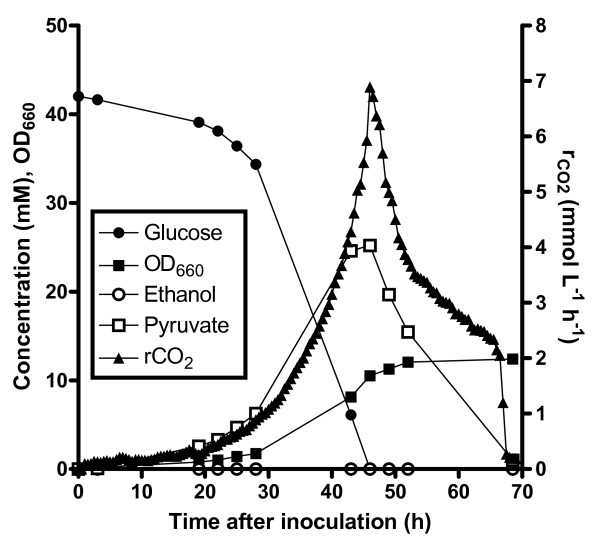
**Growth profile of IMI078 (Pdc**^**-**^***MTH1-ΔT*) on glucose as the sole carbon source.** Cultivation in an aerated pH-controlled (pH 5) bioreactor growing on 7.5 g l^-1^ glucose without external C_2_-sources. The depicted results are from one experiment of a set of four replicates, which all had identical specific growth rates (0.097 ± 0.007 h^-1^). The lag phase (rCO_2_ > 1 mmol l^-1^ h^-1^) of the replicates varied between 20 and 80 h after inoculation.

In the experiments described above, ethanol was included in the growth media to meet the requirement of Pdc^-^ strains that has been documented before and which has been attributed to a key role of pyruvate decarboxylase in the synthesis of cytosolic acetyl-coenzyme A [23-25]. Plate growth experiments indicated that both the Pdc^-^*MTH1-ΔT* strain and a Pdc^-^ strain overexpressing the wild-type *MTH1* gene grew on 20 g l^-1^ glucose without addition of ethanol as external C_2_ source (Figure 2). Although pure agarose was used in the plate experiments, a contamination with C_2_ compounds could not be entirely excluded and a further analysis was performed in aerobic, pH-controlled bioreactors on 7.5 g l^-1^ glucose without ethanol. This experiment confirmed that introduction of the *MTH1-ΔT* mutation was sufficient to enable growth in batch cultures on glucose as sole carbon source (Figure 4). In four replicate experiments a reproducible specific growth rate of 0.097 ± 0.007 h^-1^ was observed. This specific growth rate is lower than that of the evolved Pdc^-^ strain under these conditions (0.20 h^-1^) [25], suggesting that this strain may harbor additional mutations which could also contribute to the growth on glucose in the absence of added C_2_-compounds.

## Discussion

In this study the molecular basis of the ability of a *S. cerevisiae* strain evolved from a Pdc^-^ strain to grow on glucose was investigated. An internal deletion in the *MTH1* gene was identified in an evolved Pdc^-^ strain able to grow at high glucose concentrations. This internal deletion was introduced into a non-evolved Pdc^-^ strain by chromosomal integration of the identified mutation. The observation that both the evolved strain and the newly created strain have the same ability to grow on glucose supplemented with ethanol, supports the conclusion that the internal deletion within *MTH1* is solely responsible for the glucose positive growth in the evolved strain. The previously evolved Pdc^-^ strain grew without a C_2_ source with a specific growth rate of 0.20 h^-1^[25]. In this study we show that introduction of the *MTH1-ΔT* allele in the ancestral strain results in a consistent specific growth rate of 0.097 ± 0.007 h^-1^ in aerobic batch cultures on glucose as the sole carbon source. The mechanism(s) underlying the different levels of C_2_ independence between the evolved and *MTH1-ΔT* Pdc^-^ strains remains unknown. Unfortunately, the original transcriptome analysis on the evolved C_2_-source independent Pdc^-^ mutant did not provide additional clues to the mechanism [25].

*MTH1* encodes a protein that plays a critical role in the transcriptional regulation of glucose transporters in *S. cerevisiae* (for a review see [36]). Independent screens to isolate suppressors of glucose toxicity in other glycolytic mutants, such as *tpi1*[48,49,52], *pyc1 pyc2*[45], *tps1*[47], *pgk1*[46] or *pgm1* mutants [46,47] have uncovered different mutant alleles of *MTH1,* indicating its important role in glucose metabolism. A common trait of these suppressor mutants is a low glucose influx [32,45-49] that results in decreased carbon catabolite repression [32,46-49] and consequently in an increased respiration [46]. The *MTH1* alleles in these other studies carried mutations in either codon 85 or in both codon 85 and 102 [47]. These mutations are covered by the 225bp deletion spanning codons 57-131 found in the *MTH1-ΔT* allele. Two important features of Mth1 are altered in the protein resulting from the internal deletion: the alteration of a region with putative PEST sequences and the elimination of a phosphorylation site which spans from codon 118-137 [38]. PEST sequences are usually present in proteins with a short intracellular half-life [50,53]. Phosphorylation of Mth1 is also related with its degradation since only after Mth1 has been phosphorylated by Yck1 it is ubiquitinated by the SCF^grr^ complex and subsequently degraded by the proteasome [38]. Mth1 interacts with Rgt1 to repress transcription of glucose transporters and only its degradation allows their transcription. Decreased degradation of Mth1 is known to result in decreased mRNA levels for the *HXT1* gene [38]. The internal deletion of *MTH1-ΔT* shall therefore interfere with Mth1 degradation and cause a lowered expression of hexose transporters. Indeed it was found that the evolved strain presented a lowered expression of several glucose transporters [25].

The observed increase of glucose tolerance in Pdc^-^*S. cerevisiae* upon expression of a mutated allele of *MTH1* does not, in itself, explain the mechanism by which Pdc^-^ strains become glucose sensitive. Pdc^-^ strains are pyruvate hyperproducers and, in contrast to ethanol, pyruvate is unlikely to be exported by passive diffusion through the yeast plasma membrane. If it is assumed that the *MTH1* mutation mainly acts by restricting glucose transport and, therefore, glycolytic flux, it might prevent intracellular accumulation of pyruvate to toxic levels. A possible alternative explanation is related to redox metabolism. The major function of alcoholic fermentation in wild-type *S. cerevisiae* is the fast reoxidation of NADH formed in glycolysis. Unrestricted glycolytic activity in a Pdc^-^ strain, in combination with a limited capacity of the mitochondrial respiratory chain for reoxidation of cytosolic NADH, might lead to a reduction of the cytosolic NADH/NAD^+^ pool and thereby inhibit key reactions in biosynthesis.

## Conclusions

Pyruvate decarboxylase negative (Pdc^-^) *S. cerevisiae* strains are attractive metabolic engineering platforms for pyruvate-derived products, but their application was hindered by the inability to grow on high glucose concentrations and a C_2_ auxotrophy. In this study, overexpression of *MTH1* or introduction of the newly discovered allele *MTH1-ΔT* into a Pdc^-^*S. cerevisiae* strain enabled growth on glucose as the sole carbon source at industrially relevant growth rates. Furthermore, introduction of this modification partially relieves the C_2_ auxotrophy of Pdc^-^ yeasts. Therefore, *S. cerevisiae* with a disruption of the pyruvate decarboxylase genes together with expression of a more stable *MTH1* allele further increases the flexibility of *S. cerevisiae* as a platform micro-organism for the production of bio-based chemicals and fuels.

## Methods

### Strains and maintenance

Strains constructed in the present study (see Table 1) were derived from *S. cerevisiae* RWB837 [25], which contains targeted deletions of the three pyruvate decarboxylase genes *PDC1PDC5* and *PDC6* as well as a defective *URA3* allele. RWB837 was constructed in the CEN.PK background [54,55]. Strains were maintained on YP medium (demineralized water; 10 g l^-1^ yeast extract [BD Difco, Franklin Lakes, NJ, USA]; 20 g l^-1^ peptone [BD Difco]) with 2% (v/v) glycerol and 3% (v/v) ethanol. Culture stocks were prepared from shake flask cultures, which were incubated at 30°C and stirred at 200 rpm, by the addition of 20% (v/v) glycerol and were stored at -80°C.

**Table 1:** Strains used in this study

***Strain***	***Description and Genotype***	***Source***
CEN.PK113-7D	MAT**a***LEU2 URA3 MAL2-8*^*C*^	P. Kötter, Germany
RWB837	MAT**a***pdc1Δ*(-*6*,-*2*)::*loxP pdc5Δ*(-*6*,-*2*)::*loxP pdc6Δ*(-*6*,-*2*)::*loxP ura3-52*	[25]
TAM	MAT**a***pdc1Δ* (-*6*,-*2*)::*loxP pdc5Δ* (-*6*,-*2*)::*loxP pdc6Δ* (-*6*,-*2*)::*loxP ura3-52*, selected for C_2_ independence in glucose-limited chemostat cultures and glucose-tolerant growth in batch culture	[25]
MY2243	MATα *ura3-52 his3Δ1 trp1-289 pdc1Δ(-6,-2)::loxP pdc5Δ(-6,-2)::loxP*	Microbia Inc, U.S.A.
MY2280	MAT**a**/MATα *pdc1Δ/pdc1Δ pdc5Δ/pdc5Δ PDC6/pdc6Δ ura3-52/ura3-52 MTH1/MTH1-ΔT*	This study
IMI073	MAT**a***pdc1Δ*(-*6*,-*2*)::*loxP pdc5Δ*(-*6*,-*2*)::*loxP pdc6Δ*(-*6*,-*2*)::*loxP ura3-52 MTH1-ΔT::pUD143 (URA3)::MTH1*	This study
IMI076	IMI073 *ura3-52 MTH1-ΔT*	This study
IMI078	IMI076 *URA3 MTH1-ΔT*	This study
IMI082	RWB837 *URA3*	This study
IMI083	TAM *URA3*	This study
IMZ103	RWB837 pvv214	This study
IMZ104	RWB837 pEXp214-*MTH1*.2	This study

### Segregation analysis and sequencing

The evolved *S. cerevisiae* Pdc^-^ strain TAM [25] was crossed with strain MY2243 by incubating a mix of equal numbers of cells together on a YPD plate at 30°C for several hours, and selecting for robust growth on YNB plus 2% glycerol (v/v), 200 μM uracil and 2 g l^-1^ casamino acids. These conditions, under which TAM grows poorly and the other parent not at all, were used to select the diploid MY2280. Sporulation was performed by incubating a culture of MY2243 on an agar plate with 20 g l^-1^ potassium acetate and 50 μM uracil for 3-4 days at 23°C. Spores were segregated on YP agar plates supplemented with 2% ethanol (v/v) using a standard micromanipulator, incubated at 30°C and subsequently tested for growth on YP 2% agar (w/v) medium with 20 g l^-1^ glucose.

Oligonucleotides used in this study are shown in Table 2. The *MTH1* gene was amplified by PCR from genomic DNA of strains TAM and RWB837 using oligonucleotides MTH1fw and MTHrv. The PCR products were sequenced by Baseclear BV (Baseclear, Leiden, The Netherlands). The mutated *MTH1* allele of TAM was named *MTH1-ΔT*. Putative PEST regions within the Open Reading Frame of *MTH1* were identified by using the online tool ePESTfind (http://emboss.bioinformatics.nl/cgi-bin/emboss/epestfind).

**Table 2:** Oligonucleotides used in this study

***name***	***confirmation of strain***	***sequence (5'- > 3')***
**primers for cloning**		
MTH1BamHI		GCGATCGCGGATCCTTGAGGAGGTAGGGAACATC
MTH1HindIII		CTGACGCCAAGCTTAAACGGCGACTGGTGGTAAG
URA3fw		GCTGCTACTCATCCTAGTCC
URA3rv		CTTTAGCGGCTTAACTGTGC
MTH1fw		CACCATGTTTGTTTCACCACCACCAGCAACTTCG
MTH1rv		TCAGGATACTGAATCCGGCTGCCAATCCA
**primers for diagnostic PCR**		
MTH1fw	IMI073, IMI076	CACCATGTTTGTTTCACCACCACCAGCAACTTCG
MTH1rv	IMI073, IMI076	TCAGGATACTGAATCCGGCTGCCAATCCA
m13fw	IMI073, IMI076, IMZ103, IMZ104	GTAAAACGACGGCCAG
m132rv	IMI073, IMI076, IMZ103, IMZ104	GGAAACAGCTATGACCATG
URA3-A	IMI078, IMI082, IMI083	TGCATGAGTCTCAGCTCTAC
URA3-B	IMI078, IMI082, IMI083	CCAAGGGTAGAGATCCTAAG

### Plasmid and strain construction

Chromosomal introduction of the wild type *MTH1* allele in strain RWB837 was done by integrating plasmid pUD143 into the *MTH1* locus after linearization of the plasmid pUD143 by EcoRI. Construction of the plasmid pUD143 was done by amplifying the *MTH1-ΔT* allele from the evolved Pdc^-^ strain (TAM) PCR using primers MTH1bamHI and MTH1hindIII (Table 2) and cloning the fragment into plasmid pRS406 [56] by ligating HindIII and BamHI treated plasmids and inserts using T4 DNA ligase (Roche, Basel, Switzerland) to produce plasmid pUD143 (Table 3). HindIII and BamHI restriction sites were added to *MTH1-ΔT* allele by amplifying the gene from TAM genomic DNA with the primers MTH1BamHI and MTH1HindIII (Table 2) using Phusion Hot Start High Fidelity Polymerase (Finnzymes). After recombination of the plasmid pUD143 into the chromosome, which generated two copies of *MTH1*, chromosomal replacement of the wild type *MTH1* allele by the *MTH1-ΔT* allele was done using the pop-in/pop-out method [57]. A uracil auxotrophic transformant was selected on synthetic medium with trace elements and vitamins as described before [58] containing 3% (v/v) ethanol, 2% (v/v) glycerol, uracil and 5-FOA, which was named IMI076. Correct replacement of *MTH1* was confirmed by PCR using primers MTH1fw, MTH1rv, m13fw and m132rv. To obtain prototrophic strains, IMI076, RWB837 and TAM were transformed with *URA3* obtained by PCR from pRS406 [56], using Phusion Hot Start High Fidelity Polymerase (Finnzymes, Espoo, Finland). The resulting strains were named respectively IMI078, IMI082 and IMI083.

**Table 3:** Plasmids used in this study

***Plasmid***	***Characteristic***	***Reference***
pENTR/D-TOPO	Gateway entry clone	Invitrogen, USA
pvv214	2μ ori, *URA3*, P_*PGK1*_-*ccdB*-T_*CYC1*_	[59]
pENTR-D-TOPO-*MTH1*	Gateway entry clone, *MTH1*	This work
pEXp214-*MTH1*.2	2μ ori, *URA3*, P_*PGK1*_- *MTH1-ΔT*_*-TCYC1*_	This work
pRS406	Integration plasmid, *URA3*	[56]
pUD143	Integration plasmid, *URA3*, P_*MTH1*_- *MTH1-ΔT*_*-TMTH1*_	This work

Construction of plasmid pEXp214-MTH1.2 was achieved by amplification of *MTH1* from genomic DNA of CEN.PK113-7D with primers MTH1fw and MTH1rv using Phusion Hot Start High Fidelity Polymerase (Finnzymes) and subsequent cloning in pVV214 using Gateway Technology [59]. RWB837 was transformed with either pEXp214-MTH1.2 or the empty vector pVV214 resulting in strain IMZ104 and IMZ103 respectively.

Transformation and amplification of plasmids was done in *E. coli* One Shot TOP10 competent cells (Invitrogen, Grand Island, NY, USA) according to manufacturer's instructions. Plasmids were isolated from *E. coli* with the Sigma GenElute Plasmid Miniprep Kit (Sigma, St. Louis, USA).

Transformation of plasmids or linear DNA fragments in *S. cerevisiae* was done according to the lithium-based transformation protocol described by Gietz and Woods [60]. Transformants were selected on synthetic agar medium supplemented with uracil drop-out medium (Y1501, Sigma, St. Louis USA) containing 3% (v/v) ethanol and 2% (v/v) glycerol. Single colony isolates were confirmed to have the correct insert by PCR (Table 2) on colony material suspended in 0.02M NaOH and boiled for ten minutes. Plasmids were isolated from the yeast strains with Zymoprep yeast miniprep kit II (Zymo Research, Orange, CA, USA) and sequenced (Baseclear, Leiden, The Netherlands) for confirmation.

### Cultivation procedures

Cultivations were performed at 30°C in synthetic medium with glucose, trace elements and vitamins as described before [58]. Ethanol (3 ml l^-1^) was added when relevant. Cultivation on solid media was performed on medium containing 20 g l^-1^ glucose and 20 g l^-1^ of agarose (Sigma). To minimize the chance of C_2_-contamination in the medium, agarose instead of agar was chosen for the solid growth media assays. Cultivation in bioreactors was performed in medium containing 7.5 g l^-1^ glucose supplemented with Antifoam Emulsion C (Sigma), which has been autoclaved separately (120°C) as a 20% (w/v) solution and added to a final concentration of 0.2 g l^−1^.

Batch cultivations were performed in 2-liter bioreactors (Applikon, Schiedam, Netherlands) at a working volume of 1 liter. The pH was controlled by automatic addition of 2M KOH or 2M H_2_SO_4_ at a value of 5.5. Bioreactors were sparged with 500 ml min^-1^ air and stirred at 800 rpm. For growth rate measurements, the initial optical density at 660 nm (OD_660_) after inoculation was 0.1. Maximum specific growth rates were determined from duplicate cultures (errors are given as mean deviations) and were based on the OD_660_. Preculture shake flasks with synthetic medium containing 3% (v/v) ethanol were inoculated with 1 ml aliquots of frozen stock cultures. Cells from exponentially growing shake-flask precultures were washed twice with demineralized water and used to inoculate batch cultures.

### Determination of culture dry weight and optical density

Culture samples were filtered over preweighed nitrocellulose filters (pore size: 0.45 μm; Gelman Laboratory, Ann Arbor, MI). Culture dry weight was determined by weighing the filters after two washes with demineralized water and dried in a microwave oven (Bosch, Stuttgart, Germany) for 20 min at 350W. Duplicate determinations varied by less than 1%. Measurement of optical density was done at a wavelength of 660 nm in a Libra S11 spectrophotometer (Biochrom, Cambridge, UK).

### Gas and metabolite analysis

Exhaust gas was cooled in a condenser (2°C) and dried with a Permapure type MD-110-48P-4 dryer (Permapure, Toms River, NJ). Oxygen and carbon dioxide concentrations were determined with an NGA 2000 analyzer (Rosemount Analytical, Orrville, OH, USA). In calculations of rates a correction was made for sample volumes.

Glucose, ethanol and pyruvate concentrations were determined in culture supernatants with a high-performance liquid chromatography (HPLC) on a Waters Alliance 2690 HPLC (Waters, Milford, MA) containing a Bio-Rad HPX 87H column (Bio-Rad, Hercules, CA). The HPLC was operated at 60°C with 5 mM H_2_SO_4_ as mobile phase at a flow rate of 0.6 ml min^−1^. Detection was by means of a Waters 2410 refractive-index detector and a Waters 2487 UV detector.

## Competing interests

The authors declare no competing financial interests.

## Authors’ contributions

BO designed and carried out the sequencing of *MTH1*, the strain constructions, the cultivation experiments, analyzed the results and drafted the manuscript. CLF, XZ, JT and CG performed the crossing studies, identified *MTH1* as a candidate gene and revised the manuscript. JMD, JTP and AvM supervised the design, revised the manuscript and coordinated the study. All authors read and approved the final manuscript.
